# An MLST approach to support tracking of plasmids carrying OXA-48-like carbapenemase

**DOI:** 10.1093/jac/dkz136

**Published:** 2019-04-24

**Authors:** Carina Brehony, Elaine McGrath, Wendy Brennan, Alma Tuohy, Thomas Whyte, Sylvain Brisse, Martin Maiden, Keith Jolley, Dearbháile Morris, Martin Cormican

**Affiliations:** 1 Antimicrobial Resistance and Microbial Ecology Group, School of Medicine, National University of Ireland, Galway, Ireland; 2 National CPE Reference Laboratory, University Hospital Galway, Galway, Ireland; 3 Biodiversity and Epidemiology of Bacterial Pathogens, Institut Pasteur, Paris, France; 4 Department of Zoology, University of Oxford, Oxford, UK

## Abstract

**Objectives:**

The prevalence of infections caused by OXA-48-like carbapenemase-producing organisms in Ireland has increased dramatically since 2011 and is an urgent public health issue. Genome-based high-resolution genotyping was used to analyse clinical isolates submitted to the Irish Carbapenemase-Producing Enterobacteriaceae Reference Laboratory Service for a 13 month period (2016–17).

**Methods:**

A total of 109 OXA-48-producing non-duplicate clinical isolates from 16 submitting centres were sequenced. Using a gene-by-gene approach, isolate genomes were characterized by MLST and core genome MLST, and the presence of antimicrobial resistance determinants was determined. Reference mapping and a novel plasmid MLST-type approach was applied to determine plasmid background.

**Results:**

The OXA-48-like-producing isolates were *Escherichia coli* (*n* = 56), *Klebsiella* spp. (*n* = 46) and *Enterobacter cloacae* (*n* = 7). Amongst the *E. coli* isolates there were 37 different STs and amongst the *Klebsiella* spp. isolates there were 27 different STs. *bla*_OXA-48_ was present in 105/109 (96.3%) of isolates. Based on mapping analysis and detection of the pOXA-48 IncL-type plasmid replicon and backbone genes, a pOXA-48-like plasmid was identified in 93/109 isolates (85.3%). The remaining isolates (*n* = 16; 14.7%) harboured *bla*_OXA-48_-like genes in unknown environments. Using a gene-by-gene approach two pOXA-48-like plasmid groups with 2/71 pOXA-48-like locus differences between them were identified.

**Conclusions:**

In Ireland we found a diversity of genotypes associated with OXA-48-like-producing clinical isolates with the IncL pOXA-48 plasmid type predominating as the *bla*_OXA-48_ genetic environment. A plasmid MLST approach can rapidly identify plasmids associated with outbreaks and monitor spread of types temporally and geographically.

## Introduction

Carbapenems are broad-spectrum antibiotics that play a key role in therapy of infection caused by many antimicrobial-resistant Enterobacteriaceae. Global dissemination of carbapenem-resistant Enterobacteriaceae threatens the therapeutic efficacy of carbapenems. Bacteria of the Enterobacteriaceae family that produce one of a number of carbapenemase enzymes [carbapenemase-producing Enterobacteriaceae (CPE)] constitute a major public health concern. CPE have been reported in Ireland since 2009 and are detected with increasing frequency.^1^

The OXA-48-type carbapenemase first emerged in the mid-2000s in Turkey and has since been found across Europe and globally.^2–4^ CPE infections became notifiable in Ireland in 2011. Voluntary enhanced surveillance of CPE bloodstream infection was also implemented the same year. The first description of OXA-48-producing Enterobacteriaceae in Ireland (in this case *Klebsiella pneumoniae*) was in 2011 in five hospital patients.^5^ Since then the number of confirmed cases of OXA-48 has increased dramatically. In 2014, the National CPE Reference Laboratory confirmed 14 OXA-48 isolates, which increased to 274 just 2 years later.^1^ OXA-48 accounted for 77% of CPE isolates in 2016 with KPC isolates the next most prevalent at 14%. There were four OXA-48-related outbreaks reported in Irish hospitals in 2016 and almost half of the cases were attributed to one Dublin hospital experiencing an extended outbreak.^1^^,^^6^

The *bla*_OXA-48_ gene is believed to have originated on the chromosome of the environmental organism *Shewanella*.^7^ Clinical cases are predominantly associated with *K. pneumoniae* and *Escherichia coli*.^1^^,^^8^ The gene has been able to spread rapidly given its genetic environment. The gene is nested within a transposon (Tn*1999*), of which there are several variants, which is in turn predominantly carried within IncL-type plasmids 62–66 kb in size.^9^^,^^10^ Chromosomally integrated fragmented versions of the transposon have also been identified.^11^ This complexity hampers efforts to characterize bacterial isolates and also resistance element-bearing plasmids even with the availability of WGS technology. Careful characterization of isolates will help clarify the extent of the diversity of spread of these organisms and particularly the spread of transmissible elements such as plasmids.

Here we present the molecular epidemiology and genomic characterization, including a novel plasmid MLST-type approach, of OXA-48-like-producing clinical isolates submitted to the Irish National Reference Laboratory Service for CPE during a period of 13 months.

## Materials and methods

The National Reference Laboratory Service for CPE is based at University Hospital Galway and has provided reference services since October 2012. All isolates from confirmed CPE cases, detected either diagnostically from infection and/or colonization or through screening, are submitted to the CPE Reference Laboratory. The laboratory received a total of 274 OXA-48-like isolates in 2016.^1^ Of these OXA-48-like isolates 126 were from a single hospital (Hospital A) as it was experiencing an extended outbreak in this time period. Of these 126 isolates, 53 non-duplicate isolates that spanned the time period and encompassed the different species identified were selected for WGS. A further 56 non-duplicate isolates were selected to represent the diversity of other submitting hospital clinical microbiology laboratories (*n* = 15) around the country, also across the study time period. Therefore, a total of 109 non-duplicate OXA-48-like CPE isolates from individual patients from between 6 January 2016 and 31 January 2017 were analysed in this study. For all submitted isolates, initial species identification was confirmed using MALDI-TOF MS (Bruker). The MIC of meropenem was determined by Etest in accordance with EUCAST criteria.^12^ Routine phenotypic detection and characterization of suspect CPE isolates was carried out using the ROSCO KPC/MBL and OXA-48 Confirm Kit (ROSCO, Denmark). All suspect CPE isolates submitted to the laboratory were confirmed by real-time PCR for the most common genes associated with CPE (*bla*_OXA-48_, *bla*_KPC_, *bla*_NDM_, *bla*_VIM_ and *bla*_IMP_).^13^^,^^14^

### WGS and analysis

The isolates were sequenced (paired-end sequencing, read length 150 bp) using the Illumina NextSeq 500 platform (UCL Genomics, London, UK). Resulting short reads were quality checked and assembled *de novo* using Spades within the BioNumerics (Applied Maths) genomics software platform. Genomic analyses were performed using BioNumerics and the BIGSdb platform.^15^ Core genome MLST (cgMLST) was carried out within BIGSdb (https://pubmlst.org/escherichia/) using an *E. coli* cgMLST scheme downloaded from the EnteroBase database (http://enterobase.warwick.ac.uk/) and for *Klebsiella* spp. within the Pasteur *Klebsiella* BIGSdb database (http://bigsdb.pasteur.fr/klebsiella). The Genome Comparator tool in BIGSdb was used to investigate relationships amongst the isolates. This was done by carrying out pairwise allele comparisons amongst the genomes according to different sets of loci: first, cgMLST comparisons were based on 2513 loci for *E. coli* and 694 loci for *Klebsiella* spp.;^16^ and second, for plasmid comparisons using 71 loci identified in the archetypal pOXA-48 plasmid (JN626286) (Table S1, available as Supplementary data at *JAC* Online). The two IS*1999* transposase genes in JN626286 were excluded from the pOXA-48 MLST analysis due to the difficulty in assembly of short reads for these genes. Other pOXA-48 plasmid reference sequences used in the plasmid MLST analysis, for comparison purposes, were pRA35 (LN864821), pE71T (KC335143) and pKpn-E1.Nr7 (KM406491).^9^^,^^17^^,^^18^*Klebsiella* spp. reference genomes were used for comparison with *Klebsiella* phylogroups.^19^ Distance matrices generated were used to create NeighborNet diagrams using Splitstree version 4.14.2.^20^ The GrapeTree plugin in BIGSdb was used to create a minimum-spanning tree of the pOXA-48 locus differences amongst isolates.^21^ The ResFinder database was used to identify plasmid replicon types and antibiotic resistance-associated genes (https://cge.cbs.dtu.dk/services/ResFinder).^22^ The *Enterobacter cloacae* seven-locus MLST scheme hosted on the PubMLST database was used for speciation and to obtain ST (http://pubmlst.org/ecloacae). Species identification for *Klebsiella* spp. isolate genomes was carried out using the rMLST.org website based on exact matches to ribosomal protein gene alleles.^23^ MEGA version 7.0 was used for sequence alignment.^24^ The National Center for Biotechnology Information website (http://www.ncbi.nlm.nih.gov/BLAST/) was used for sequence queries.

For plasmid reference mapping analysis, short reads were mapped against known complete plasmids: pOXA-48 (JN626286, 61.8 kb) and pOXA-181 (KP400525, 51 kb).^25^^,^^26^ Short-read reference mapping and processing was carried out using SMALT version 0.7.4 and Samtools version 0.1.11.^27^^,^^28^ Resulting processed mapped reads and annotated reference sequences were visualized using Artemis version 16.0.0.^29^

## Results

### Characterization of OXA-48-like isolates

**Figure 1.**
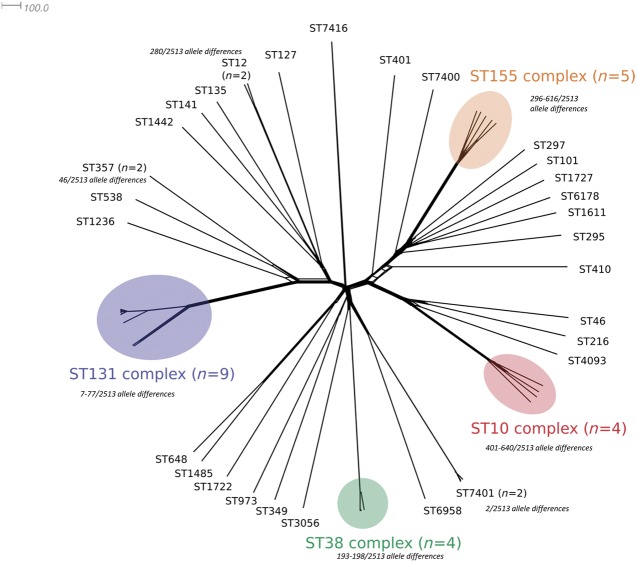
NeighborNet diagram based on pairwise allele comparisons of core genome locus alleles (*n* = 2513) amongst the genomes of OXA-48 *E. coli* isolates (*n* = 56) in Ireland. STs and major clonal complexes are indicated. The scale bar represents the number of locus allele differences. Allele differences between closely related isolates are indicated. This figure appears in colour in the online version of *JAC* and in black and white in the print version of *JAC*.

**Figure 2.**
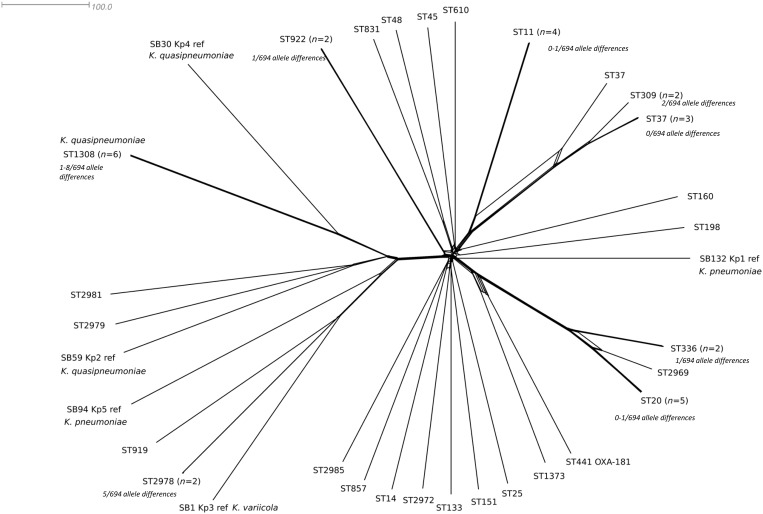
NeighborNet diagram based on pairwise allele comparisons of core genome locus alleles (*n* = 694) amongst the genomes of OXA-48 *Klebsiella* spp. isolates (*n* = 46) in Ireland. STs and reference isolate genomes of *Klebsiella* spp. phylogroups are indicated. The scale bar represents the number of locus allele differences. Allele differences between closely related isolates are indicated.

There were a total of 56 *E. coli*, 46 *Klebsiella* spp. and 7 *E. cloacae* isolates analysed. Within the 46 *Klebsiella* spp. isolates there were 35 *K. pneumoniae*, 8 *Klebsiella quasipneumoniae* and 3 *Klebsiella variicola* isolates identified (Table S2). There was a genotypically diverse set of seven-locus MLST STs within each species (Table 1 and Figures 1 and 2). Amongst the *E. coli* isolates, there were 37 STs (Achtman scheme), of which ST131 was the most prevalent (*n* = 9, 16.1%) followed by ST38 (*n* = 4) and ST10 (*n* = 3). Amongst the *Klebsiella* spp. isolates there were 27 STs, of which *K. quasipneumoniae* ST1308 was the most common (*n* = 6, 13.0%) followed by ST20 and ST11. Amongst the *E. cloacae* isolates there were four ST66 isolates and one each of ST108, ST110 and ST135.

**Table 1.** Genotyping of *bla*_OXA-48_-like-harbouring isolates from Ireland (*n = *109)

Species	Number of isolates	OXA-48-like alleles (number)	STs (number)^a^	Plasmid replicons	β-Lactamase genes (number)	Other resistance genes
*E. coli*	56	OXA-48 (55), OXA-244 (1)	131 (9), 38 (4), 10 (3), 12 (2), 58 (2), 155 (2), 357 (2), 1049 (2), 7401 (2)	IncL, IncFII, IncX1, ColRNAI, Col156, Col, Col8282	*bla*_TEM-1_ (16), *bla*_CTX-M-24_ (3), *bla*_CTX-M-15_ (2), *bla*_CTX-M-14_ (2), *bla*_CTX-M-9_ (1), *bla*_CTX-M-3_ (1), *bla*_OXA-1_ (1), *bla*_CMY-2_ (1), *bla*_DHA-1_ (1)	*aac(3)-Iia*, *aac(3)-Iid*, *aac(6')Ib-cr*, *aadA1*, *aadA2*, *aadA5*, *catA1*, *catB3*, *dfrA1*, *dfrA12*, *dfrA14*, *dfrA17*, *erm*(B), *mph*(A), *qnrB4*, *qnrS1*, *strA*, *strB*, *sul1*, *sul2*, *tet*(A)
*Klebsiella* spp.	46	OXA-48 (44), OXA-181 (2)	1308 (6), 20 (5), 11 (4), 37 (4), 309 (2), 336 (2), 922 (2), 2978 (2)	IncL, IncX3, IncR, ColRNAI, ColKP3	*bla*_CTX-M-15_ (14), *bla*_CTX-M-14_ (1), *bla*_TEM-1_ (6), *bla*_SHV-1_ (6), *bla*_SHV-11_ (12), *bla*_SHV-27_ (1), *bla*_SHV-164_ (1), *bla*_SHV-38_ (1), *bla*_NDM-5_ (1), *bla*_ACC-1_ (1), *bla*_LEN12_ (2), *bla*_LEN24_ (1), *bla*_OKP-A-8_ (2), *bla*_OKP-A-8_ (1), *bla*_OKP-B-2_ (1)	*aac(3)-Iia*, *aac(3)-Iid*, *aac(6')Ib-cr*, *aadA1*, *aadA2*, *aadA5*, *aph(3')-Ia*, *ARR-3*, *catA1*, *catA2*, *catB3*, *dfrA1*, *dfrA12*, *dfrA14*, *dfrA17*, *floR*, *fosA*, *mph*(A), *oqxA*, *oqxB*, *qnrB66*, *qnrB7*, *qnrS1*, *strA*, *strB*, *sul1*, *sul2*, *tet*(A), *tet*(D)
*E. cloacae*	7	OXA-48 (6), OXA-181 (1)	66 (4), 108 (1), 110 (1), 135 (1)	IncL, TrfA, IncX3, IncR, ColRNAI	*bla*_OXA-1_ (6), *bla*_CTX-M-15_ (4), *bla*_CTX-M-9_ (3), *bla*_SHV-12_ (1), *bla*_TEM-1_ (1), *bla*_ACT-7_ (5), *bla*_ACT-14_ (1), *bla*_ACT-15_ (1)	*aacA4*, *aac(3)-IIa*, *aac(6')Ib-cr*, *aadA1*, *aadA2*, *aadB*, *catA1*, *catB3*, *dfrA14*, *dfrA16*, *fosA*, *mph*(A), *qnrA1*, *qnrB1*, *strA*, *strB*, *sul1*, *sul2*, *tet*(A)

a Only STs with number >1 are given for *E. coli* and *Klebsiella* spp.

cgMLST schemes for *E. coli* and *K. pneumoniae* were used to determine genetic relationships amongst isolates (Figures 1 and 2). There were between 2 and 2495 out of 2513 core genome locus differences amongst *E. coli* genomes. One hospital (Hospital A), which contributed 48.6% (*n* = 53) of the isolates, had a diverse set of bacterial genotypes that appeared to be circulating over the time period. In Hospital A, amongst the *E. coli* isolates (*n* = 33) there were 26 different STs and amongst the *Klebsiella* spp. isolates (*n* = 20) there were 12 different STs. Some of the major *E. coli* lineages were found across all sites, but some were more localized, suggestive of local transmission. For example, *E. coli* ST131 and ST38 were found distributed throughout the country, while *K. quasipneumoniae* ST1308 and *K. pneumoniae* ST20 were only found in Hospitals A and B, respectively.

The *bla*_OXA-48_ gene was present in 105/109 (96.3%) of isolates, while the remaining 4 isolates harboured *bla*_OXA-181_ [*K. pneumoniae* (*n = *1), *K. quasipneumoniae* (*n = *1) and *E. cloacae* (*n = *1)] and *bla*_OXA-244_ [*E. coli* (*n = *1)]. One *K. pneumoniae* isolate (id 5493, Pasteur *Klebsiella* BIGSdb database) harboured a *bla*_NDM-5_ gene as well as *bla*_OXA-48_. Many of the isolates also contained genes encoding ESBLs (Table 1). A total of nine *E. coli* isolates (16.1%) harboured a *bla*_CTX-M_ gene [*bla*_CTX-M-24_ (*n = *3), *bla*_CTX-M-15_ (*n = *2), *bla*_CTX-M-14_ (*n = *2), *bla*_CTX-M-9_ (*n = *1) and *bla*_CTX-M-3_ (*n = *1)]. A total of 15 *Klebsiella* spp. isolates (32.6%) harboured a *bla*_CTX-M_ gene variant [*bla*_CTX-M-15_ (*n = *14) and *bla*_CTX-M-14_ (*n = *1)]. All *E. cloacae* isolates harboured a *bla*_CTX-M_ gene [*bla*_CTX-M-9_ (*n = *3) and *bla*_CTX-M-15_ (*n = *4)]. For the three *bla*_OXA-181_-harbouring isolates [*Klebsiella* spp. (*n = *2) and *E. cloacae* (*n = *1)] the quinolone resistance gene *qnrS1* was found on the same assembled contig sequence and therefore the same plasmid as *bla*_OXA-181_.

### Genomic context of OXA-48-like genes

A variety of plasmid replicon types were detected, including those of Inc-type plasmids (L, X1, X3, R, IncFII) and Col-type plasmids (RNAI, KP3, 156, 8282) (Table 1). A total of 93/109 isolates (85.3%) harboured the replicon and three backbone genes characteristic of the archetypal IncL-type pOXA-48 plasmid, *repA*, *traU* and *parA*.^25^ Of the isolates with *bla*_OXA-48_ but without the IncL-type plasmid replicon (*n = *11), two (both *E. coli*) did not contain *repA*, *traU* and *parA*, and nine contained just *parA* [*E. coli* (*n = *8) and *K. pneumoniae* (*n = *1)]. No plasmid replicons were found in four isolates (3.7%, all *E. coli*) that harboured *bla*_OXA-48_. These four isolates contained the same 29 of the 71 loci identified in the reference pOXA-48 plasmid.

Read mapping to plasmid reference sequences was carried out for all isolates. Of the 93 harbouring *repA*, *traU* and *parA*, and the IncL replicon, all except 1 mapped to the whole length of the ∼63 kb reference pOXA-48 (JN626286) plasmid sequence. They harboured all the other 71 pOXA-48 loci. The exception (id 113, *E. coli* BIGSdb) appeared to be truncated and only mapped to a stretch of ∼51 kb of the pOXA-48 reference sequence as well as harbouring just 51/71 pOXA-48 loci (loci consecutive). Another isolate genome (id 144, *E. coli* ST12) containing the IncL pOXA-48 replicon but which was missing the *traU* locus appeared to be truncated and contained 39/71 pOXA-48 loci (loci consecutive). Mapping revealed a region of the reference sequence ∼28 kb long to which short reads did not map. The unmapped region included the plasmid mobilization genes *mobA* and *mobB* and plasmid transfer protein genes *traH* to *traU*. The three *bla*_OXA-181_-harbouring isolates mapped to the 51 kb pOXA-181 IncX3-type plasmid reference sequence (KP400525). Three of the isolates’ plasmid genes were identical at all 62 loci of the KP400525 pOXA-181 reference plasmid, while the fourth isolate differed at 4 loci (two transposases, a type IV secretion protein gene and a plasmid replication gene).

All nine isolates harbouring only *parA* but harbouring *bla*_OXA-48_ [*E. coli* (*n* = 8) and *K. pneumoniae* (*n* = 1)] mapped to an ∼23 kb consecutive region of the reference pOXA-48 (JN626286) sequence (Table S1). This region spanned from the IS*1999delta*-IS*1* at the upstream part of the transposon and the second IS*1* element after *korC* and *orf*25. The isolates spanned the time period of the study and were from eight different submitting centres. The isolates were eight *E. coli* (ST131, 38, 12, 69, 12) and one *K. pneumoniae* (ST11). All nine isolates contained the same 29/71 pOXA-48 loci (Table S1). The *bla*_OXA-244_ isolate (id 104, *E. coli* ST1722) also mapped to an ∼23 kb region of the pOXA-48 plasmid and harboured the same 29/71 loci.

One isolate (id 97, *E. coli* ST410) appeared to have *bla*_OXA-48_ integrated into a Tn*1999.3* variant within a Col-type plasmid as the Col replicon and *bla*_OXA-48_ were on the same assembled contig sequence. A further isolate (id 101, *E. coli* ST38) also appeared to have a truncated part of the pOXA-48 plasmid as short reads mapped only to an ∼7 kb region in the pOXA-48 reference sequence around the Tn*1999* element from the IS*1999* element downstream of OXA-48 and upstream to *mucA*. Isolates id 97 and id 101 harboured just 3 and 4 of 71 pOXA-48 loci, respectively, including *bla*_OXA-48_ itself. Isolates id 97 and id 101 had Col and IncF replicons detected, respectively, but no IncL replicon.

### Plasmid molecular epidemiology using pOXA-48 gene-by-gene approach

**Figure 3.**
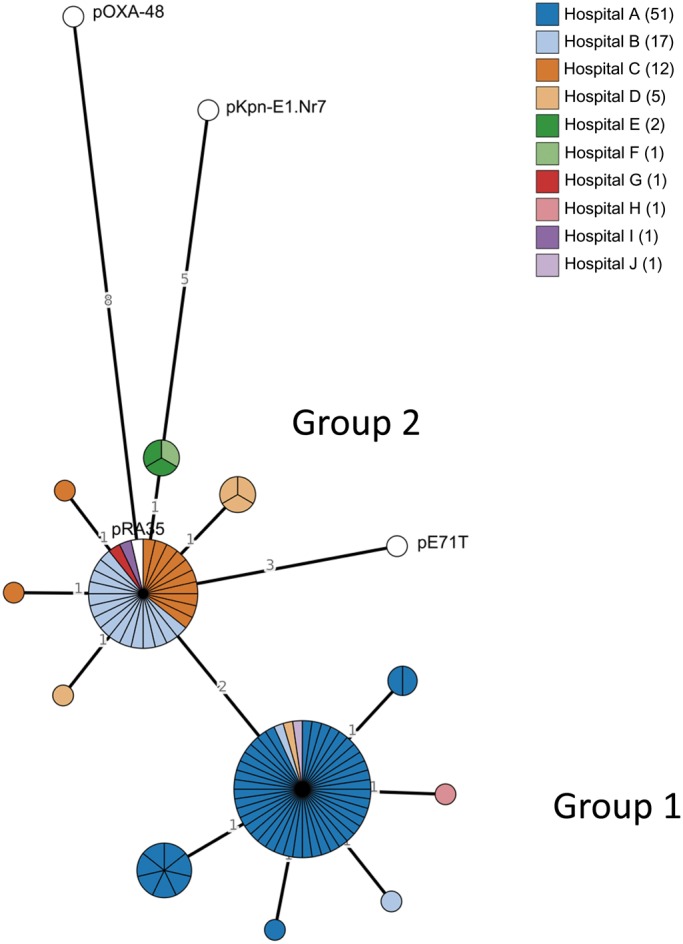
Minimum-spanning tree of pOXA-48 plasmid (JN626286) locus (*n* = 71) allele differences amongst isolates with an IncL pOXA-48-type plasmid (*n* = 92). Each circle (node) contains isolates with identical profiles at the 71 locus alleles. Lines (edges) connecting nodes indicate the number of locus allele differences between nodes. Nodes are divided in pie-chart form for individual isolates. Nodes are coloured by isolate-submitting hospital. Four complete reference plasmids (coloured white in nodes) were used in the analysis for comparison: pOXA-48 (JN626286), pRA35 (LN864821), pE71T (KC335143) and pKpn-E1.Nr7 (KM406491). The minimum-spanning tree was constructed using the GrapeTree plugin in BIGSdb. This figure appears in colour in the online version of *JAC* and in black and white in the print version of *JAC*.

There were two groupings of pOXA-48 plasmid types based on pOXA-48 MLST allele profiles (Figure 3). A total of 44 isolates (47.8%) within the first group (Group 1) and 27 isolates (29.3%) in the second group (Group 2) were identical at 69 loci of the pOXA-48 plasmid reference (JN626286). These two groups differed from each other at two loci; one was a hypothetical protein (*orf*10 in pOXA-48 annotation) and the other a putative restriction endonuclease (*orf*16 in pOXA-48 annotation). Isolate plasmids of Group 2 were identical at all 71 loci with the pRA35 plasmid. No isolate plasmids were identical at all 71 loci to the reference pOXA-48 plasmid.

Each group had isolates from the whole time span of the collection and were represented by at least three species (Figure S1). Each group contained *E. coli* and *K. pneumoniae* isolates, but Group 1 contained all *K. quasipneumoniae* and *K. variicola* isolates, while all *E. cloacae* were within Group 2. The majority (41/44) of isolates from Group 1 were from Hospital A, while the majority of Group 2 were from Hospital B (15/25), which indicates some local homogeneity of plasmid types in particular within Hospital A. Group 2 had a more diverse geographical spread with isolates encompassing (if including the single locus variants of the main cluster) all four provinces of the country.

## Discussion

The number of patients colonized with CPE detected in Ireland has increased dramatically since 2012 (*n* = 5). In 1 year between 2015 and 2016 the increase was 156% (139 to 356 cases), though it is likely that increased ascertainment is one factor contributing to the apparent increase.^1^ An outbreak in one major hospital has contributed a high proportion (48.6%) of the isolates studied.

In common with several other countries, in Ireland the *bla*_OXA-48_ gene was associated with several species and with several lineages or clonal groups within each species. There were, however, two groups of pOXA-48-like plasmids and a number of variants related to these groups. One of these plasmid types was the predominant *bla*_OXA-48_ carrier in a large hospital with an extended outbreak. The variety of species and genotypes that bore this plasmid in this case leads to characterization of this as a ‘plasmid outbreak’. This is an important distinction, as failure to recognize that CPE isolates of different species may be linked by carriage of a common plasmid may lead to delay in recognizing an outbreak.

While the IncL-type pOXA-48 plasmid was the predominant carrier for the carbapenemase gene, there was an indication that the gene was integrated within a pOXA-48 plasmid fragment with an unknown genetic background for a number of isolates (*n* = 12). This would have to be verified with long-read sequencing using PacBio SMRT or Minion technologies to span the whole region. However, previous studies have shown that the gene can be maintained chromosomally at a low level in the population.^9–11^^,^^30^ Most of the isolates we examined with the pOXA-48 plasmid fragment belonged to globally distributed extraintestinal pathogenic *E. coli* lineages ST131, ST38 and ST127, in which the gene has been identified chromosomally previously.^9^^,^^11^^,^^31^ One of the isolates possessed the single peptide variant *bla*_OXA-244_ (*E. coli*).^30^ As with plasmid-associated OXA-48, there does not appear to be a single bacterial clone associated with these OXA-48 isolates with unknown genetic background, as several clones disseminated throughout Ireland. The range of plasmid types associated with *bla*_OXA-48_ appears to be widening, including truncated and much larger versions of the IncL-type pOXA-48 plasmid as well as other plasmid family types.^32–34^ In Ireland there were a small number of isolates that appeared to have either truncated IncL-type plasmids or other plasmid types such as Col. Again it must be noted that gene-by-gene approaches and mapping analysis are hindered in their ability to detect multiple copies of similar plasmids and rearrangements within and between them. Therefore, long-read sequencing should be used to bring further clarity to the chromosomal and accessory genome contexts.

The dissemination of *bla*_OXA-48_ has been aided by the particular propensity of the pOXA-48 IncL-type plasmid for horizontal genetic transfer (HGT) and within-host transfer of the plasmid amongst Enterobacteriaceae species is likely to occur since co-colonization and infection with multiple species harbouring the gene is known.^10^^,^^35^^,^^36^ It is likely that in a Catch 22-like scenario, colonization is prolonged by HGT and HGT is aided by prolonged colonization.^37^ Duration of colonization with OXA-48-producing organisms lasts for at least several months, increasing the likelihood of spread within the healthcare environment and within the community.^37^^,^^38^ While carriage prevalence of OXA-48-producing organisms in the community is as yet not well studied, in Western Europe it is currently thought to be low.^39^^,^^40^ However, this is likely to change over time with the apparent increase in this CPE in Europe from its original and now endemic reservoir in the Near and Middle East and North Africa.^41^

Hospital outbreaks of OXA-48 are becoming more common in Ireland and elsewhere as organisms carrying the plasmid may spread in hospital from individuals who are colonized/infected.^5^^,^^42–44^ A critical element in the control of an outbreak or epidemic is recognition of the outbreak and an understanding of the underlying biology. This work contributes to the growing acceptance that the traditional paradigm of an outbreak or epidemic as a phenomenon characterized by a series of closely related isolates is not always applicable. This paradigm is not applicable to outbreaks and epidemics of acquired antimicrobial resistance encoded on highly mobile genetic elements that transmit to several lineages and species. Rapid genomic analysis such as the gene-by-gene MLST-type approach to plasmids applied here can support rapid characterization and improved focus on tracking the mobile genetic elements that are the actual driver of the outbreak.

## Supplementary Material

dkz136_Supplementary_Data
