# Supplementary material for: An MLST approach to support tracking of plasmids carrying OXA-48-like carbapenemase
Source: J Antimicrob Chemother. 2019 Apr 24;74(7):1856–62. doi: 10.1093/jac/dkz136 (PMC6587408; doi:10.1093/jac/dkz136)

**Supplementary data**

**Table S1.** Loci of reference plasmid pOXA-48 (JN626286) used in plasmid MLST analysis (n=71)

| JN626286 locus name | *E. coli* db/Enterobase locus id | locus present in ~23kb pOXA-48 plasmid fragment |
| --- | --- | --- |
| orf11 | A4R38_RS24790 | yes |
| orf10 | A4R38_RS24795 | yes |
| repA | A4R38_RS24905 |  |
| excA | A4R38_RS24915 |  |
| traX | A4R38_RS24925 |  |
| mobB | A4R38_RS25020 |  |
| orf30 | A4R38_RS25060 |  |
| orf29 | A4R38_RS25065 |  |
| trbB | A4R39_RS24495 |  |
| trbC | A4R39_RS24500 |  |
| repC | A4R39_RS24510 |  |
| traW | A4R39_RS24530 |  |
| traU | A4R39_RS24535 |  |
| orf38 | A4R39_RS24540 |  |
| traR | A4R39_RS24545 |  |
| traQ | A4R39_RS24550 |  |
| traP | A4R39_RS24555 |  |
| traO | A4R39_RS24560 |  |
| traN | A4R39_RS24565 |  |
| traM | A4R39_RS24570 |  |
| traJ | A4R39_RS24600 |  |
| traI | A4R39_RS24605 |  |
| traH | A4R39_RS24610 |  |
| mobA | A4R39_RS24615 |  |
| mobC | A4R39_RS24625 |  |
| orf34 | A4R39_RS24630 |  |
| orf33 | A4R39_RS24635 |  |
| ssb | A4R39_RS24640 |  |
| orf32 | A4R39_RS24645 |  |
| orf31 | A4R39_RS24650 |  |
| klcA | A4R39_RS24655 |  |
| orf27 | A4R39_RS24705 |  |
| orf26 | A4R39_RS24710 |  |
| ccgA1 | A4R39_RS24715 |  |
| korC | A4R39_RS24720 | yes |
| orf23 | A4R39_RS24730 | yes |
| orf22 | A4R39_RS24735 | yes |
| orf21 | A4R39_RS24740 | yes |
| orf20 | A4R39_RS24745 | yes |
| orf18 | A4R39_RS24775 | yes |
| orf17 | A4R39_RS24780 | yes |
| orf14 | A4R39_RS24795 | yes |
| orf12 | A4R39_RS24800 | yes |
| orf9 | A4R39_RS24820 | yes |
| orf8 | A4R39_RS24825 | yes |
| orf7 | A4R39_RS24830 | yes |
| orf6 | A4R39_RS24835 | yes |
| orf5 | A4R39_RS24840 | yes |
| blaOXA-48 | A4R39_RS24880 | yes |
| lysR | A4R39_RS24885 | yes |
| trbN | A4R39_RS24905 |  |
| parB | ECE24377A_RS00610 | yes |
| parA | ECE24377A_RS00615 | yes |
| orf24 | hypothetical_A4R39 | yes |
| orf16 | hypothetical_pOXA_588 | yes |
| orf36 | hypothetical_pOXA_666 |  |
| mucA | mucA | yes |
| mucB | mucB | yes |
| nuc | Nuc | yes |
| orf19 | orf19 | yes |
| orf25' | orf25' |  |
| orf28 | orf28 |  |
| orf67 | orf67 |  |
| pemI | pemI | yes |
| pemK | pemK | yes |
| repB | repB |  |
| resD | resD | yes |
| rmoA | rmoA |  |
| traL | traL |  |
| traY | traY |  |
| trbA | trbA_full |  |

**Table S2.** Details of OXA-48 isolate genomes from Ireland used in study (n=109). SLV=single locus variant

| **BIGSdb id *E. coli*/*Klebsiella* spp.** | **ENA Accession** | **ST** | **species** | **referring Hospital** | **pOXA-48 plasmid group** | ***repA*, *traU*, *parA*** | **mapping** |
| --- | --- | --- | --- | --- | --- | --- | --- |
| 118 | ERR1837602 | 10 | *E. coli* | A | 1 | all present | pOXA-48 type |
| 125 | ERR1987530 | 10 | *E. coli* | D | group 2 SLV | all present | pOXA-48 type |
| 108 | ERR2124244 | 10 | *E. coli* | A | 1 | all present | pOXA-48 type |
| 5432 | ERR1813604 | 11 | *K. pneumoniae* | F | group 2 SLV | all present | pOXA-48 type |
| 5438 | ERR1813609 | 11 | *K. pneumoniae* | E | group 2 SLV | all present | pOXA-48 type |
| 5439 | ERR1813610 | 11 | *K. pneumoniae* | E | group 2 SLV | all present | pOXA-48 type |
| 5503 | ERR2118035 | 11 | *K. pneumoniae* | L |  | parA | pOXA48 fragment |
| 105 | ERR1987537 | 12 | *E. coli* | A |  | parA | pOXA48 fragment |
| 144 | ERR2124237 | 12 | *E. coli* | A |  | repA and parA | pOXA48 fragment |
| 5500 | ERR2109180 | 14 | *K. pneumoniae* | C | 2 | all present | pOXA-48 type |
| 5425 | ERR1811832 | 20 | *K. pneumoniae* | B | 2 | all present | pOXA-48 type |
| 5431 | ERR1813603 | 20 | *K. pneumoniae* | B | 1 | all present | pOXA-48 type |
| 5433 | ERR1813605 | 20 | *K. pneumoniae* | B | 2 | all present | pOXA-48 type |
| 5434 | ERR1813606 | 20 | *K. pneumoniae* | B | 2 | all present | pOXA-48 type |
| 5444 | ERR1830484 | 20 | *K. pneumoniae* | B | 2 | all present | pOXA-48 type |
| 5486 | ERR2118023 | 25 | *K. pneumoniae* | A | 1 | all present | pOXA-48 type |
| 5423 | ERR1810608 | 37 | *K. pneumoniae* | A | 1 | all present | pOXA-48 type |
| 5443 | ERR1815293 | 37 | *K. pneumoniae* | B | 2 | all present | pOXA-48 type |
| 5460 | ERR1829917 | 37 | *K. pneumoniae* | B | 2 | all present | pOXA-48 type |
| 5461 | ERR1829918 | 37 | *K. pneumoniae* | B | 2 | all present | pOXA-48 type |
| 120 | ERR1981374 | 38 | *E. coli* | C | 2 | all present | pOXA-48 type |
| 100 | ERR1981375 | 38 | *E. coli* | J |  | parA | pOXA48 fragment |
| 101 | ERR1837604 | 38 | *E. coli* | C |  | none | pOXA48 fragment |
| 114 | ERR2124249 | 38 | *E. coli* | N |  | parA | pOXA48 fragment |
| 5472 | ERR1840990 | 45 | *K. pneumoniae* | D | group 2 SLV | all present | pOXA-48 type |
| 145 | ERR2124238 | 46 | *E. coli* | A | 1 | all present | pOXA-48 type |
| 5493 | ERR2118025 | 48 | *K. pneumoniae* | B | 2 | all present | pOXA-48 type |
|  | ERR2107336 | 66 | *E. cloacae* | B | 2 | all present | pOXA-48 type |
|  | ERR2124550 | 66 | *E. cloacae* | C | 2 | all present | pOXA-48 type |
|  | ERR2124552 | 66 | *E. cloacae* | C | group 2 SLV | all present | pOXA-48 type |
|  | ERR2124553 | 66 | *E. cloacae* | C | 2 | all present | pOXA-48 type |
| 148 | ERR1990257 | 69 | *E. coli* | F |  | parA | pOXA48 fragment |
| 152 | ERR1837600 | 101 | *E. coli* | A | 1 | all present | pOXA-48 type |
|  | ERR2124555 | 108 | *E. cloacae* | C |  | none | pOXA-181 type |
|  | ERR2107337 | 110 | *E. cloacae* | G | 2 | all present | pOXA-48 type |
| 102 | ERR1981376 | 127 | *E. coli* | K |  | parA | pOXA48 fragment |
| 99 | ERR1981603 | 131 | *E. coli* | B | 1 | all present | pOXA-48 type |
| 98 | ERR1981606 | 131 | *E. coli* | P |  | parA | pOXA48 fragment |
| 119 | ERR1981373 | 131 | *E. coli* | D | group 2 SLV | all present | pOXA-48 type |
| 103 | ERR1981378 | 131 | *E. coli* | P |  | parA | pOXA48 fragment |
| 131 | ERR1987773 | 131 | *E. coli* | A | 1 | all present | pOXA-48 type |
| 140 | ERR2124233 | 131 | *E. coli* | A | 1 | all present | pOXA-48 type |
| 107 | ERR2124243 | 131 | *E. coli* | C | 2 | all present | pOXA-48 type |
| 112 | ERR2124247 | 131 | *E. coli* | A | 1 | all present | pOXA-48 type |
| 115 | ERR2124250 | 131 | *E. coli* | B |  | parA | pOXA48 fragment |
| 5429 | ERR1811833 | 133 | *K. pneumoniae* | A | 1 | all present | pOXA-48 type |
| 135 | ERR1990255 | 135 | *E. coli* | A | 1 | all present | pOXA-48 type |
|  | ERR2124556 | 135 | *E. cloacae* | C | 2 | all present | pOXA-48 type |
| 113 | ERR2124248 | 141 | *E. coli* | O | 2 | all present | pOXA48 fragment |
| 5504 | ERR2118036 | 151 | *K. pneumoniae* | B | 2 | all present | pOXA-48 type |
| 128 | ERR1987538 | 155 | *E. coli* | D | 1 | all present | pOXA-48 type |
| 142 | ERR2124235 | 155 | *E. coli* | A | group 1 SLV | all present | pOXA-48 type |
| 5468 | ERR1829922 | 160 | *K. pneumoniae* | C | 2 | all present | pOXA-48 type |
| 5428 | ERR1811836 | 198 | *K. pneumoniae* | C | 2 | all present | pOXA-48 type |
| 137 | ERR2124230 | 216 | *E. coli* | A | 1 | all present | pOXA-48 type |
| 99 | ERR1837603 | 295 | *E. coli* | A | 1 | all present | pOXA-48 type |
| 151 | ERR2124251 | 297 | *E. coli* | A | 1 | all present | pOXA-48 type |
| 5498 | ERR2118029 | 309 | *K. pneumoniae* | B | 2 | all present | pOXA-48 type |
| 5499 | ERR2118030 | 309 | *K. pneumoniae* | B | 2 | all present | pOXA-48 type |
| 5480 | ERR1840996 | 336 | *K. pneumoniae* | A | 1 | all present | pOXA-48 type |
| 5502 | ERR2118032 | 336 | *K. pneumoniae* | A | group 1 SLV | all present | pOXA-48 type |
| 133 | ERR1987775 | 349 | *E. coli* | A | group 1 SLV | all present | pOXA-48 type |
| 110 | ERR2124245 | 357 | *E. coli* | B | 2 | all present | pOXA-48 type |
| 111 | ERR2124246 | 357 | *E. coli* | B | 2 | all present | pOXA-48 type |
| 149 | ERR1987531 | 401 | *E. coli* | A | 1 | all present | pOXA-48 type |
| 97 | ERR1981602 | 410 | *E. coli* | F |  | none | Col type |
| 5487 | ERR2118024 | 441 | *K. pneumoniae* | M |  | none | pOXA-181 type |
| 127 | ERR1987534 | 538 | *E. coli* | A | group 1 SLV | all present | pOXA-48 type |
| 5454 | ERR1830483 | 610 | *K. pneumoniae* | B | group 1 SLV | all present | pOXA-48 type |
| 150 | ERR1987540 | 648 | *E. coli* | C | group 2 SLV | all present | pOXA-48 type |
| 5451 | ERR1830485 | 834 | *K. pneumoniae* | A | 1 | all present | pOXA-48 type |
| 5476 | ERR1840993 | 857 | *K. pneumoniae* | A | group 1 SLV | all present | pOXA-48 type |
| 5469 | ERR1840988 | 919 | *K. variicola* | A | 1 | all present | pOXA-48 type |
| 5467 | ERR1829921 | 922 | *K. pneumoniae* | A | 1 | all present | pOXA-48 type |
| 5475 | ERR1840992 | 922 | *K. pneumoniae* | A | 1 | all present | pOXA-48 type |
| 123 | ERR2124227 | 973 | *E. coli* | A | 1 | all present | pOXA-48 type |
| 130 | ERR1987772 | 1049 | *E. coli* | A | group 1 SLV | all present | pOXA-48 type |
| 132 | ERR1987774 | 1049 | *E. coli* | A | group 1 SLV | all present | pOXA-48 type |
| 109 | ERR2118033 | 1236 | *E. coli* | A | 1 | all present | pOXA-48 type |
| 5465 | ERR1829919 | 1308 | *K. quasipneumoniae* | A | 1 | all present | pOXA-48 type |
| 5466 | ERR1829920 | 1308 | *K. quasipneumoniae* | A | 1 | all present | pOXA-48 type |
| 5471 | ERR1840989 | 1308 | *K. quasipneumoniae* | A | 1 | all present | pOXA-48 type |
| 5473 | ERR1840991 | 1308 | *K. quasipneumoniae* | A | 1 | all present | pOXA-48 type |
| 5477 | ERR1840994 | 1308 | *K. quasipneumoniae* | A | group 1 SLV | all present | pOXA-48 type |
| 5491 | ERR2109179 | 1308 | *K. quasipneumoniae* | A | 1 | all present | pOXA-48 type |
| 5481 | ERR1840997 | 1373 | *K. pneumoniae* | A | group 1 SLV | all present | pOXA-48 type |
| 141 | ERR2124234 | 1442 | *E. coli* | A | group 1 SLV | all present | pOXA-48 type |
| 134 | ERR1990253 | 1485 | *E. coli* | A | 1 | all present | pOXA-48 type |
| 147 | ERR2124240 | 1611 | *E. coli* | H | group 1 SLV | all present | pOXA-48 type |
| 104 | ERR1987532 | 1722 | *E. coli* | D |  | parA | pOXA-244 fragment |
| 129 | ERR1987539 | 1727 | *E. coli* | A | 1 | all present | pOXA-48 type |
| 5484 | ERR1841001 | 2969 | *K. pneumoniae* | A | 1 | all present | pOXA-48 type |
| 5437 | ERR1813608 | 2972 | *K. pneumoniae* | D | group 2 SLV | all present | pOXA-48 type |
| 5488 | ERR2109181 | 2978 | *K. variicola* | A | 1 | all present | pOXA-48 type |
| 5489 | ERR2109158 | 2978 | *K. variicola* | A | 1 | all present | pOXA-48 type |
| 5507 | ERR2109157 | 2979 | *K. quasipneumoniae* | C |  | none | pOXA-181 type |
| 5427 | ERR1811835 | 2981 | *K. quasipneumoniae* | J | 1 | all present | pOXA-48 type |
| 5501 | ERR2118031 | 2985 | *K. pneumoniae* | I | 2 | all present | pOXA-48 type |
| 124 | ERR2124228 | 3056 | *E. coli* | A | 1 | all present | pOXA-48 type |
| 121 | ERR1837605 | 4093 | *E. coli* | C | 2 | all present | pOXA-48 type |
| 116 | ERR2124252 | 6178 | *E. coli* | C | 2 | all present | pOXA-48 type |
| 106 | ERR2124242 | 6958 | *E. coli* | A | 1 | all present | pOXA-48 type |
| 126 | ERR1987535 | 7400 | *E. coli* | A | 1 | all present | pOXA-48 type |
| 136 | ERR1987533 | 7401 | *E. coli* | A | 1 | all present | pOXA-48 type |
| 138 | ERR2124231 | 7401 | *E. coli* | A | 1 | all present | pOXA-48 type |
| 122 | ERR1981379 | 7416 | *E. coli* | A | 1 | all present | pOXA-48 type |
| 143 | ERR2124236 | 7696 | *E. coli* | A | 1 | all present | pOXA-48 type |
| 139 | ERR2124232 | 58(155cc) | *E. coli* | A | 1 | all present | pOXA-48 type |
| 146 | ERR2124239 | 58(155cc) | *E. coli* | A | 1 | all present | pOXA-48 type |

**Figure S1.** Minimum-spanning tree of pOXA-48 plasmid (JN626286) locus (n=71) allele differences amongst isolates with IncL pOXA-48 type plasmid (n=92) by species.  Each circle (node) contains isolates with identical profiles at the 71 locus alleles. Lines (edges) connecting nodes indicate the number of locus allele differences between nodes. Nodes are divided in pie-chart form for individual isolates. Nodes are coloured by isolate species. The minimum-spanning tree was constructed using the GrapeTree plugin in BIGSdb.


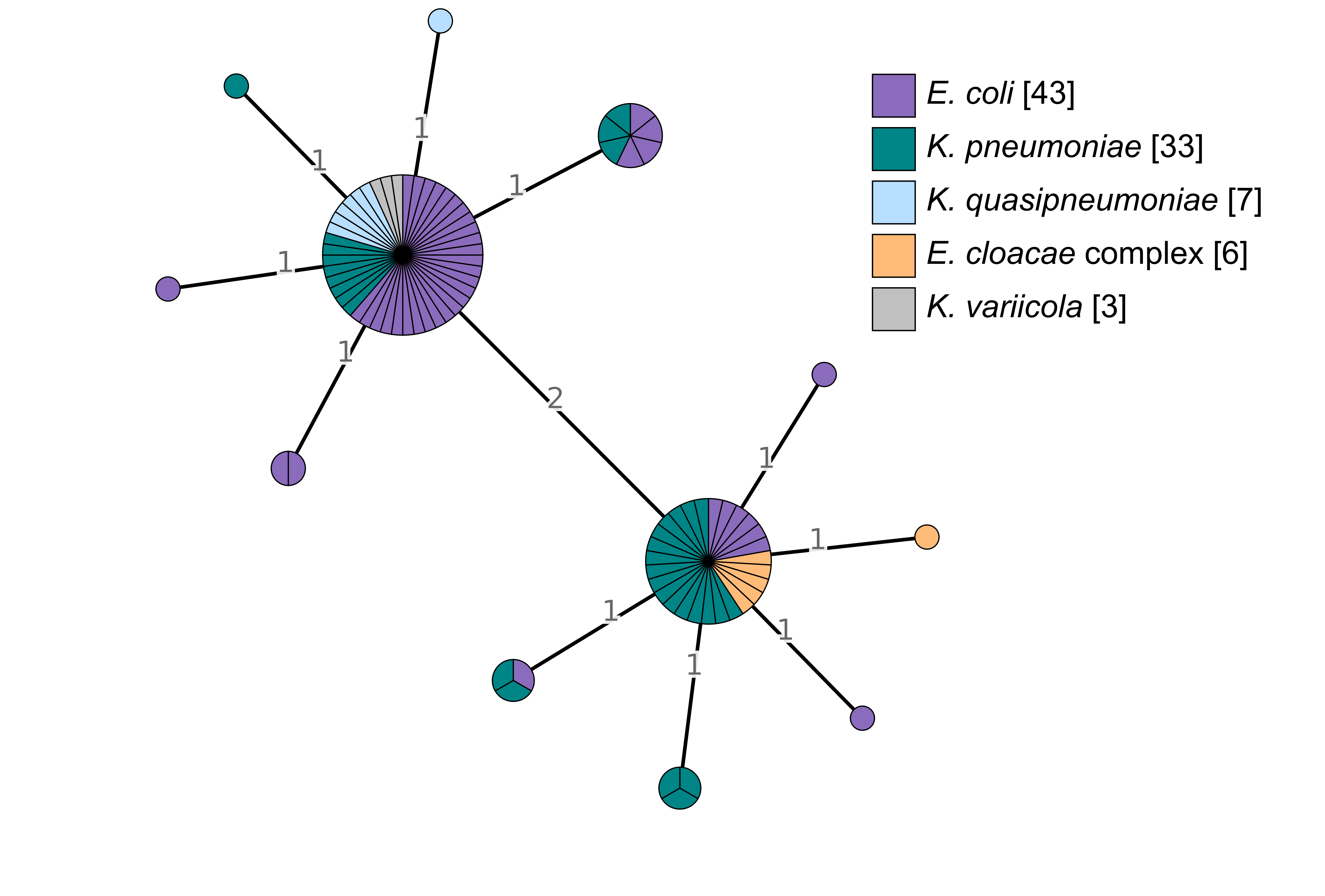

Supplement: dkz136_Supplementary_Data [file dkz136_supplementary_data.docx]
